# Explorative Characterization of GI Complaints, General Physical and Mental Wellbeing, and Gut Microbiota in Trained Recreative and Competitive Athletes with or without Self-Reported Gastrointestinal Symptoms

**DOI:** 10.3390/nu16111712

**Published:** 2024-05-30

**Authors:** Floris C. Wardenaar, Alex E. Mohr, Carmen P. Ortega-Santos, Jean Nyakayiru, Christine Kersch-Counet, Yat Chan, Anna-Marie Clear, Jonathan Kurka, Kinta D. Schott, Ryan G. N. Seltzer

**Affiliations:** 1 College of Health Solutions, Arizona State University, Phoenix, AZ 85004, USA; aemohr@asu.edu (A.E.M.); ychan19@asu.edu (Y.C.); aclear@asu.edu (A.-M.C.); jonathan.kurka@asu.edu (J.K.); kschott3@asu.edu (K.D.S.); ryan.seltzer@asu.edu (R.G.N.S.); 2 Center for Health Through Microbiomes, Biodesign Institute, Arizona State University, Tempe, AZ 85281, USA; 3 Department of Exercise and Nutrition Sciences, Milken Institute School of Public Health, The George Washington University, Washington, DC 20052, USA; carmen.ortegasantos@email.gwu.edu; 4 FrieslandCampina, 3818 LE Amersfoort, The Netherlands; jean.nyakayiru@frieslandcampina.com (J.N.); christine.kersch@frieslandcampina.com (C.K.-C.)

**Keywords:** fermented whey, multi-ingredient, supplement, healthy, *Bifidobacterium*

## Abstract

The current state of the literature lacks a clear characterization of gastrointestinal (GI) symptoms, gut microbiota composition, and general physical and mental wellbeing in well-trained athletes. Therefore, this study aimed to characterize differences in self-reported symptoms, gut microbiota composition, and wellbeing (i.e., sleep quality, mood, and physical (PHQ) and mental wellbeing) between athletes with and without GI symptoms. In addition, we assessed the potential impact of a 3-week multi-ingredient fermented whey supplement in the GI complaints group, without a control group, on the gut microbiota and self-reported GI symptoms and wellbeing. A total of 50 athletes (24.7 ± 4.5 years) with GI issues (GI group at baseline, GI-B) and 21 athletes (25.4 ± 5.3 years) without GI issues (non-GI group, NGI) were included. At baseline, there was a significant difference in the total gastrointestinal symptom rating scale (GSRS) score (24.1 ± 8.48 vs. 30.3 ± 8.82, *p* = 0.008) and a trend difference in PHQ (33.9 ± 10.7 vs. 30.3 ± 8.82, *p* = 0.081), but no differences (*p* > 0.05) were seen for other outcomes, including gut microbiota metrics, between groups. After 3-week supplementation, the GI group (GI-S) showed increased *Bifidobacterium* relative abundance (*p* < 0.05), reported a lower number of severe GI complaints (from 72% to 54%, *p* < 0.001), and PHQ declined (*p* = 0.010). In conclusion, well-trained athletes with GI complaints reported more severe GI symptoms than an athletic reference group, without showing clear differences in wellbeing or microbiota composition. Future controlled research should further investigate the impact of such multi-ingredient supplements on GI complaints and the associated changes in gut health-related markers.

## 1. Introduction

As the recipient of nutrients, the gastrointestinal (GI) tract plays an important role in athleticism. From long-term considerations of exercise recovery to enhanced exercise-induced adaptations to the immediate aspects of acute bouts of effort during competition, the entire GI system must work in concert with an individual to achieve performance outcomes. However, GI distress is widely prevalent among athletes, ranging from 30 to 90% in recreationally and professionally active athletes (e.g., bloating and diarrhea) [1,2]. Recent data have suggested that athletes tend to report, more often, gastrointestinal symptoms in general (i.e., 62% in rugby players and 61% in American football players) than during training or competition (i.e., 47% and 50%, respectively) [3,4]. Although still poorly understood, factors such as exercise intensity and duration, demographic factors (e.g., sensitivity to certain carbohydrates), and environmental factors (e.g., heat stress) have been suggested to play a role [5,6,7,8]. Furthermore, the widespread use of nutritional supplements and specialized dietary regimens among athletes adds complexity to the issue [9,10,11]. For example, it is important to consider that food and fluid intake during exercise may impact GI symptoms and beyond. Although training the gut by consuming a high level of carbohydrates daily and during training reduces GI complaints during exercise, it may still result in higher gastrointestinal rating scores than a more moderate consumption of carbohydrates, hence influencing the self-reported complaints of some athletes [12].

The gut microbiota (i.e., the microbes that cohabit the human GI tract) is an important determinant of GI function [13], including gut barrier integrity and structure [14,15], and an alteration in its composition has been suggested as a contributor to exercise-associated GI symptoms in athletes [16]. The literature has suggested that the hypoxia-reperfusion experienced in the gut during high-intensity endurance exercise could negatively impact gut microbiota by reducing ecological diversity and the ratio of commensal-to-pathogen taxa (i.e., dysbiosis), consequently disrupting the gut barrier by increasing the permeability of the gut epithelium [17,18,19]. Compromising the GI epithelium can lead to GI symptoms, such as increased bowel movement and reduced gastric emptying, bloating, and diarrhea [20], ultimately reducing general wellbeing [1]. Yet, the exact effects of exercise on gut microbiota disruption, GI permeability, and GI symptoms in athletes remain poorly understood. Hence, more insight into differences on multiple physiological levels between those reporting and not reporting GI complaints is warranted.

To address exercise-associated GI symptoms and restore gut health in athletes, a healthy diet supplemented with probiotic organisms and fermentable soluble fiber, such as resistant starch and vegetables, has been proposed [21]. Such a multi-ingredient approach may allow GI symptoms to be addressed by targeting various metabolic and signaling pathways [22,23,24]. Recent studies have suggested that prebiotics can enhance gut colonization by beneficial bacteria such as *Bifidobacterium* [25,26,27], improve bacterial fermentation, control gut permeability and inflammation, and alleviate GI symptoms [9,28,29]. Furthermore, the fermentation of dairy has been shown to not only alter the digestion and/or absorption kinetics of dairy proteins [30], but to give rise to the presence of potentially bioactive metabolites [31,32,33]. The ingestion of such fermented products may reduce the prevalence of GI complaints by stimulating positive changes in several gut-health related disturbances [34,35]. Interestingly, the use of gut microbiota-altering ingredients in sports supplements has also been associated with other areas of health, including improvements in sleep [36], mood and stress [37], and overall wellbeing [38]. Due to the suggested stress and/or sleep-related origin of certain GI complaints [25,39], athletes experiencing GI complaints may, therefore, consider exploring a variety of nutritional supplements, such as prebiotic, probiotic, or synbiotic products, to support their GI and overall health.

This study aimed to explore the relationship between GI symptoms, wellbeing, and gut microbiota associated with an active lifestyle in well-trained athletes. The primary objective was to characterize better GI symptom rating scores, gut microbiota composition, and general physical and mental wellbeing between athletes with and without self-reported GI symptoms. The second objective was to explore the potential impact over time of a multi-ingredient fermented whey supplement on the previously mentioned outcomes, only provided to the group of athletes with GI complaints.

## 2. Materials and Methods

### 2.1. Study Design

**Figure 1:**
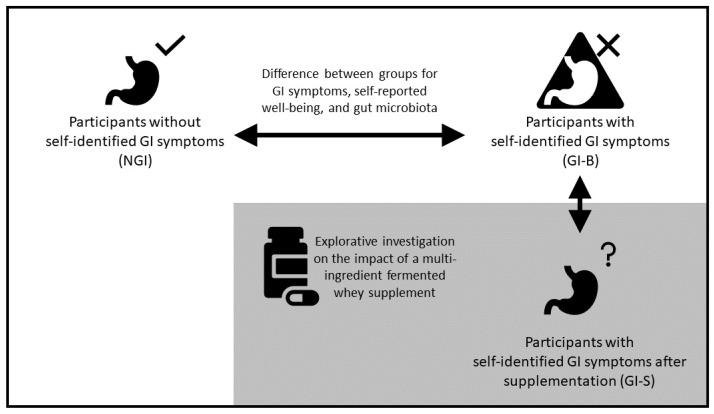
Study design. The upper part describes the comparison between athletes without self-identified gastrointestinal symptoms (NGI) and athletes with GI symptoms (GI-B) at baseline, followed by the comparison between GI-B and GI-S after supplementing a multi-ingredient fermented whey supplement. The arrows indicate the direction of assessment over time on self-reported gastrointestinal symptoms ratings, gut microbiota composition, dietary intake, self-reported wellbeing, sleep, and mood states.

The study consisted of an observational design to compare well-trained athletes at baseline with GI symptoms (GI group, GI-B) and without symptoms (non-GI group, NGI), as shown in Figure 1. During this 3-week observational period, all participants consumed their own protein supplement as normal without any intervention, but GI complaints, wellbeing, and gut microbiota were assessed. In addition, also shown in Figure 1, a single-arm intervention study was performed, in which the outcome of GI-B was compared to the outcome of a 3-week supplementation phase with a multi-ingredient fermented whey supplement with 8 g of protein and 4 g of soluble fiber (galactooligosaccharides), i.e., the intervention supplement (GI-S). Participants were asked to maintain their normal usage of protein supplements within two hours after practice, but replace 8 g of their normal supplement with the intervention supplement. In case they did not consume a protein supplement after training, subjects were asked to consume the intervention supplement before going to bed, ensuring daily consumption of the supplement. The primary outcome was the assessment of self-reported gastrointestinal symptoms ratings (GSRSs) between the GI-B and NGI groups; secondary outcomes included the gut microbiota, dietary intake, self-reported physical and mental wellbeing, sleep, and mood disturbance. For this purpose, measurements were taken on day 1 and day 22 of the study and then averaged. Additionally, an explorative analysis was also performed to assess the potential impact of the intervention supplement during a 3-week period, comparing the outcome of GI-B (based on the average of day 1 and day 22) with GI-S (measured at day 43). All demographics, self-reported questionnaires, and body composition measurements, except for the 24 h dietary recalls, were recorded using REDCap (Vanderbilt University, Nashville, TB, USA).

### 2.2. Study Participants

**Figure 2:**
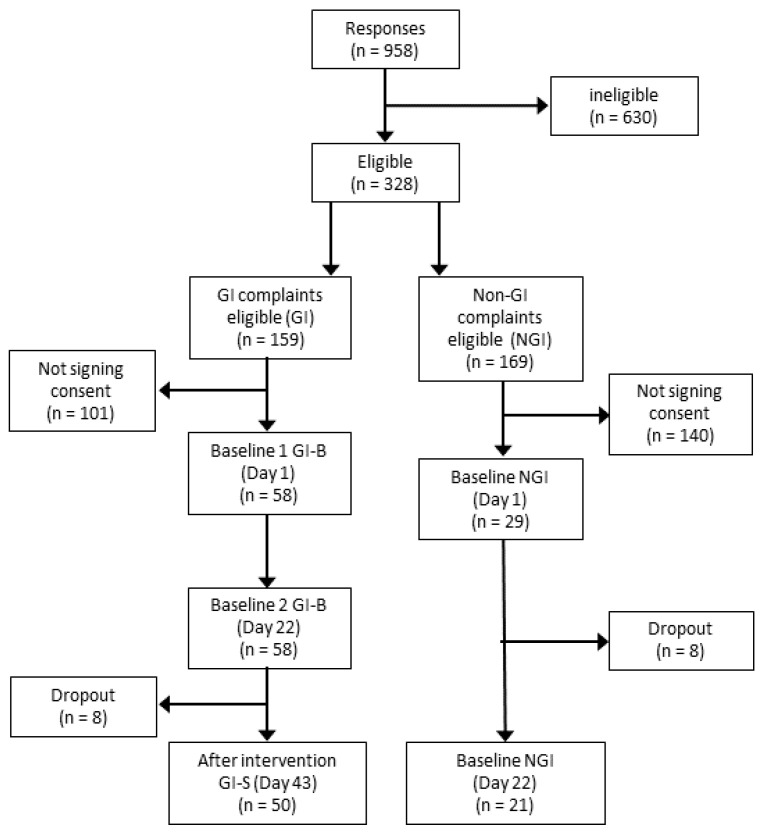
Consort diagram. The figure shows the number of recreational athletes interested in participating in the study, as well as the number that was eligible and signed informed consent, followed by dropout and the final number of participants finishing the study.

Study participants were recruited via direct mailings and via social media ads targeting adult frequent exercisers in the Phoenix area, AZ (USA). We included healthy-weight, stable male and female athletes training at least three times per week, aged 18–35 years old, with self-identified GI symptoms as part of GI-B and GI-S (50 subjects) and athletes without self-identified GI symptoms in NGI (21 subjects), as shown in Figure 2. Additional inclusion criteria were body mass index (BMI) in the range of 18.5–40.0 kg/m^2^, no physical limitations, regular use of protein supplements or sports food(s) containing protein at least three times per week, and the willingness to slightly increase protein supplement intake during the intervention phase for 21 consecutive days when being part of the GI group. Exclusion criteria included the use of probiotics and/or prebiotic supplements, smoking, self-reported cow’s milk protein allergy, or being diagnosed with: lactose intolerance, a milk protein allergy, or house dust mite allergy. Those using medications known to affect protein metabolism (i.e., corticosteroids, non-steroidal anti-inflammatories, or prescribed acne medications), gastric acid-suppressing medication or anti-coagulants, as well as antibiotics or anti-inflammatory medication within the past two weeks or blood donation within the past two months, or those being pregnant were also excluded. The study was approved by the Arizona State Institutional Review Board (STUDY00014399), with ClinicalTrials.gov ID NCT05303753, and all subjects provided written informed consent.

The sample size was powered to detect a change in GI symptom ratings over time. Based on previous work [40], reporting a significant median difference in GI symptoms after probiotic supplementation resulted in a score of 4 with a range of 0–25 at baseline vs. a score of 2 with a range of 0–16 after supplementation (*p* < 0.010). We used the formula $X=\frac{\left(a+2m+b\right)}{4}$, where “m” is the median and “a” represents the low end, and “b” represents the high end of the range [41], to estimate the mean and standard deviation for both data points. This estimation resulted in a Cohen’s D effect size of 0.51. Using this effect size, we calculated a minimal sample size for the GI group of n = 27 while considering an α of 0.05 and power of 0.80 (GPower 3.1) and focusing on a larger range of measurements than GI symptoms alone. Assuming that the NGI group only would report no to a very limited number of GI complaints, and having no clear reference data for this group, no minimal sample size was determined for the NGI group before the start of the study, and the investigators aimed to include as many participants as possible within the time frame of the study.

### 2.3. Dietary Intervention: Multi-Ingredient Supplement

Due to the exploratory nature of the study, the intervention product, a multi-ingredient supplement, was provided for only 3 weeks to the GI group after the 3-week comparative period with NGI. Delivered in a sachet, each 15 g serving of the supplement (Biotis^®^ Fermentis, provided by FrieslandCampina, Amersfoort, The Netherlands) provided 5 g carbohydrate, 8 g protein, and 0.5 g fat (1418 kJ/100 g), and contained the following main ingredients: whey protein concentrate and galactooligosaccharides fermented with *Lactobacillus rhamnosus* GG (LGG) and yogurt cultures.

### 2.4. Assessment of Body Composition

At the start of the study, instruction videos and measurement tapes were provided allowing participants to record body height, waist circumference, and hip circumference (cm), as well as body weight (kg). These self-reported measurements, as a result of COVID-19 restrictions during data collection, were used to calculate the BMI (kg/m^2^) and waist-to-hip ratio (waist circumference/hip circumference). 

### 2.5. Measurement of Digestive Symptoms

The subjects’ digestive comfort was measured using the GSRS questionnaire, previously tested for validity and reliability [42,43,44]. The GSRS is a 15-item self-administered questionnaire divided into five clusters of symptoms: reflux, abdominal pain, indigestion, diarrhea, and constipation. These symptoms are graded on a 7-point Likert scale, where 1 signifies no discomfort at all and 7 represents very severe discomfort. The total score has been calculated, as well as the percentage of subjects reporting symptoms (≥2), or moderate to severe symptoms (≥4).

### 2.6. Analysis of Gut Microbiota

#### 2.6.1. Fecal Sample Collection and Storage

Participants received instructions and videos for at-home fecal sample collection using swabs containing a DNA/RNA shield reagent (R1101, Pangea Laboratory, Tustin, CA, USA), ensuring stability at room temperature. The samples were then transferred at 2–3 days after collection to the ASU College of Health Solutions, Phoenix, AZ, USA. The samples were kept frozen at −20 °C until overnight shipment to BaseClear B.V. (Leiden, The Netherlands) for analysis. 

#### 2.6.2. DNA Extraction and Analysis Methods

DNA was extracted from fecal samples using the ZymoBIOMICS 96 MagBead DNA Kit (Zymo Research, Irvine, CA, USA). Subsequent DNA quantitation involved the Quant-iT™ dsDNA Broad-Range Assay Kit (Quant-iT™, Invitrogen, Waltham, MA, USA) and agarose gel electrophoresis for DNA integrity. The DNA template underwent PCR amplification with primers 341F and 785R, generating a ~630 bp amplicon [45]. This targeted the V3–V4 regions of the 16S rRNA gene for subsequent next-generation sequencing on Illumina MiSeq. Unique Index Primers were added in a second PCR cycle, and the resulting products were purified using Agencourt© AMPure^®^ XP (Becker Coulter, Brea, CA, USA). Subsequently, PCR amplicons were equimolarly pooled, followed by sequencing on an Illumina MiSeq with the paired-end 300 cycles protocol and indexing. FASTQ read sequence files were generated using bcl2fastq version 2.20 (Illumina, San Diego, CA, USA). The initial quality assessment was based on data passing the Illumina Chastity filtering. PhiX control signal-containing reads were removed through an in-house filtering protocol. Additionally, (partial) adapter-containing reads were clipped, ensuring a minimum read length of 50 bp. The remaining reads underwent a second quality assessment using FASTQC tool version 0.11.8. Paired-end sequence reads were collapsed into so-called pseudo-reads using sequence overlap with USEARCH version 9.2 [46]. Pseudo-reads were taxonomically classified based on alignment results with SNAP version 1.0.23 [47] against RDP database [48] version 11.5 for bacterial organisms.

#### 2.6.3. Microbiota Composition Outcomes

Paired-end, demultiplexed data were imported and analyzed using QIIME 2 (v 2021.8) software and run through DADA2 to remove low-quality regions and construct a feature table using amplicon sequence variants (ASVs). Next, the ASV feature table was passed through a weighted classifier using q2-clawback [49], pre-trained to discern taxonomy mapped to the latest version of the rRNA database SILVA (138.1; 99% OTUs from the 515F/806R region of sequences) [50]. A phylogenic tree was then constructed using the fragment-insertion plugin with the SILVA database. A rarefaction threshold was assessed at 8700 and used to impute high-quality reads and normalize for uneven sequencing depths between samples [51]. A *phyloseq* (v1.38.0) object was created, and downstream analyses and visualizations were performed in R (v.4.1.2). Sequences were removed, including mitochondrial, plant DNA, and singleton ASVs, across all samples. Alpha diversity was calculated using the Shannon index, Chao1 Index, and phylogenetic diversity (PD). Beta-diversity was assessed using phylogenetically informed qualitative (weighted UniFrac distances) and quantitative (unweighted UniFrac distances) metrics.

### 2.7. Measurements of Physical and Mental Wellbeing, Sleep Quality, and Total Mood Disturbance

#### 2.7.1. Physical Wellbeing

The validated Physical Health Questionnaire (PHQ) covers sleep disturbance, headache, GI problems, and respiratory infections using 14 items with Likert scales scoring in the range of 1–7, from “not at all” to “all the time”. The total score is in the range of 14–98, where a low score indicates better physical health [52].

#### 2.7.2. Mental Wellbeing

The validated Kessler Psychological Distress Scale (K6) addresses depressed mood, motor agitation, fatigue, worthless guilt, and anxiety. The K6 uses a 0–3 Likert scale, ranging from “none of the time” to “all of the time”. Total score is in the range of 0–18, where a low score indicates low mental distress, with ≥13 suggesting mental distress [53].

#### 2.7.3. Sleep Quality

The validated 8-item Athens Insomnia Scale (AIS) was used to assess the subjects’ sleep quality. Scores in the range of 0–3 indicate “no problems at all” to “very serious problems” [54]. The total score will be 0–24, where a score of 0 represents no sleeping issues and a score of 24 represents very serious sleeping problems. A score ≥6 is considered to represent athletes having sleeping issues [54]. 

#### 2.7.4. Total Mood Disturbance

The validated 36-item short Profile of Mood States (POMS) questionnaire estimates the intensity of mood disturbance. A higher score indicates a disturbed mood and a lower score indicates less of a disturbance. This results in a value between –24 and 177, with lower scores indicative of people with more stable mood profiles [55].

### 2.8. Assessments of Dietary Intake and Food Quality

Subjects were asked before the start of the study not to make any changes to their diet for the duration of the study. To determine the stability of dietary intake and food quality, three 24 h recalls and three food frequency questionnaires were collected on days 1, 22, and 43, analyzed using the Automated Self-Administered 24 h (ASA24) Dietary Assessment Tool, version (ASA24-2020), developed by the National Cancer Institute, Bethesda, MD, USA [56], providing insights into energy, macronutrient, and fiber intakes. 

The Rapid Eating Assessment for Participants (REAP) questionnaire was used to assess food quality over a 3-week period. Twenty-five nutrition questions from the REAP questionnaire were included and scored as usually/often (1), sometimes (2), or rarely/never (3), resulting in a total score of 25–75, with higher scores indicating healthier eating behavior, as previously described by our research group [57]. 

### 2.9. Statistics

Data were checked for normality using histograms and assessments of skewness and kurtosis; the majority of the questionnaire-based outcomes were not normally distributed. Demographics and question-based results have been reported as mean ± standard deviation and median with interquartile range (IQR) for total and stratified groups. Mixed models were used to test the differences in outcome measures between groups at baseline and over time with baseline (only for the GI group). The comparison for the supplement intervention was run using random coefficient models, with the time period of product use specified as a time-dependent covariate. This overall model structure allowed for the estimation of changes in the slope of outcomes throughout the study. Linear mixed models were run on continuous outcome variables and total survey scores. Generalized linear mixed models were used for not normally distributed categorical variables, and when needed, relevant covariates, such as sex and waist–hip ratio (WHR), were included. Both mixed models specified the subject and the time period as random effects. Significance was set at *p* ≤ 0.05, but due to the exploratory nature of this study, *p*-values ≤ 0.10 were considered trend significant. Additionally, Eta-Squared was calculated as $\eta^{2}=\frac{SSeffect}{SStotal}$ to examine effect sizes (small = 0.01–<0.08, medium = 0.08–0.26, large = >0.26 [58].

For the gut microbiota, log-transformed alpha diversity (Shannon index, Chao1, and PD) metrics were assessed with linear mixed models, testing the effect of time and the interaction of group and time using the nLME package in R (v3.1-162). Beta-diversity was compared with permutational multivariate analysis of variance (PERMANOVA; permutation n = 999) using the “adonis” function in the vegan package (v2.6.2). The PERMANOVA model incorporated the factors of time and interaction (group × time). Additionally, PERMANOVA models were constructed for all 25 components of the REAP questionnaire to assess the influence of diet on the structure of the gut microbiota. To compare dispersion, a permutation test for homogeneity in multivariate dispersion (PERMDISP) was conducted using the “betadisper” function in the vegan package. Beta-diversity distances were also compared between groups [59] by calculating the within-subject distance for paired samples (day 1 vs. day 22 and combined baseline (day 1 and day 22) vs. day 43) and testing for group distances (Wilcoxon rank-sum test). For differential abundance testing between groups and time, analysis of compositions of microbiomes with bias correction was employed on taxa present in at least 20% of all the samples using the R package ANCOMBC (v1.4.0) [60]. The covariates of sex, WHR, and REAP scores were used in all the above models. Finally, based on an a priori hypothesis that *Bifidobacterium* abundance could be affected by the study product, *Bifidobacterium* was independently assessed for the GI group at baseline and after supplementation at day 43. Specifically, we performed a log2 transformation and performed a Kruskal–Wallis test with pairwise comparisons. Adjustment for multiple testing was conducted with a Benjamini–Hochberg correction (q-value < 0.05).

## 3. Results

### 3.1. Demographics

The analysis included data from n = 21 NGI- and n = 50 GI-group athletes after completion of data collection. Although the NGI (48%) group seemed to have slightly more females than the GI-B (32%) group, this was not significantly different (χ^2^ (1) = 1.410, *p* = 0.235). The difference in age between groups (*p* = 0.60), with similar body composition (*p* ≥ 0.25), is displayed in Table 1. The majority of subjects were non-Hispanic White males, with the NGI group including significantly more Asian/Asian American subjects than GI-B (29% vs. 10%, *p* = 0.05). Both groups had a similar number of strength athletes, but NGI included fewer endurance athletes than GI-B (14% vs. 42%, *p* = 0.02), which was compensated by NGI with more other-sports types (24% in NGI vs. 6% in GI-B, *p* = 0.010).

The REAP score, energy and protein intake as g, g/kg, and percentage of kcal delivered by protein as en% as assessed by ASAP24 showed no significant differences between groups (*p* ≥ 0.114; Table 2). However, significant differences were observed between groups in absolute protein intake (157 ± 98.7 vs. 101 ± 46.7 g, *p* = 0.022), protein intake in g/kg (1.96 ± 0.80 vs. 1.42 ± 0.44 g/kg, *p* = 0.021), and fiber intake (22.2 ± 10.2 vs. 16.4 ± 6.60 g/kg, *p* = 0.036).

### 3.2. GI Complaints and Wellbeing

As shown in Table 2, the total GSRS was six points lower for NGI (24.1 ± 8.48) compared to GI-B (30.3 ± 8.82) at baseline (*p* = 0.008), followed by a trend-significant higher score for PHQ (*p* = 0.081) at baseline between NGI (33.9 ± 10.7) and GI-B (30.3 ± 8.82). The other secondary measurements shown in Table 2, such as mental (K6) wellbeing, sleep quality (AIS-8), and total mood disturbance (POMS), were not significantly different between groups at baseline (*p* ≥ 0.497). Parsing out the 15 individual constituents of the GSRS, NGI reported a score of 1 (no complaints) more frequently compared to GI-B (Table 3). However, some subjects in NGI also reported scores of 5 and 6, in the range of 5–10% for individual items. In contrast, participants in GI-B more frequently reported scores of 5 and 6, in the range of 4–16, with a higher incidence of a score of 6. Bloating and gas/flatus were most often scored as severe complaints in both groups, but subjects reported a wide variety of complaints ranging from moderate to severe. When classifying the outcomes for GI-B, 72% of subjects reported at baseline at least one severe complaint, which was after supplementation reduced for GI-S to 54% (χ^2^ (4) = 24.2, *p* < 0.001).

### 3.3. Gut Microbiota Assessment

After three weeks of supplementation with the intervention product, there was a trend that GSRS was lower in the GI-S compared to the GI-B group (*p* = 0.076). For GI-S, the PHQ score was significantly lower than GI-B (day 1: 38.5 ± 9.68 vs. day 43: 33.3 ± 10.7, *p* = 0.010), indicating improved physical wellbeing. The other secondary outcomes, such as K6 (*p* = 0.295), AIS-8 (*p* = 0.230) and total mood disturbance (*p* = 0.595), were not altered from baseline to day 43.

We obtained n = 19 (NGI) and n = 47 (for both GI-B and GI-S) complete stool sample sets for the gut microbiota assessment. To assess the baseline stability of the gut microbiota, we first compared NGI and GI-B on day 1 and day 22. We did not note any significant shifts in the alpha (*p* ≥ 0.543; Supplementary Figure S1A–C) or beta (*p* ≥ 0.782; Supplementary Tables S1 and S2) diversity metrics, taxonomic composition (q ≤ 0.05; Supplementary Figure S1F), or intra-subject distances (*p* ≥ 0.359; Supplementary Figure S1D,E) surveyed. Tests of homogeneity of dispersion did not reveal any significant differences (*p* ≥ 0.331), increasing our confidence that no baseline differences were found between groups and our findings were not an artifact of variance in group or time dispersion. Interestingly, upon testing associations of alpha diversity metrics to relevant covariates, we did find significant negative correlations between exercise volume (hours per week) and Chao1 (Spearman rho = −0.238, q = 0.005) and PD (Spearman rho = −0.326, q = 0.0001).

**Figure 3:**
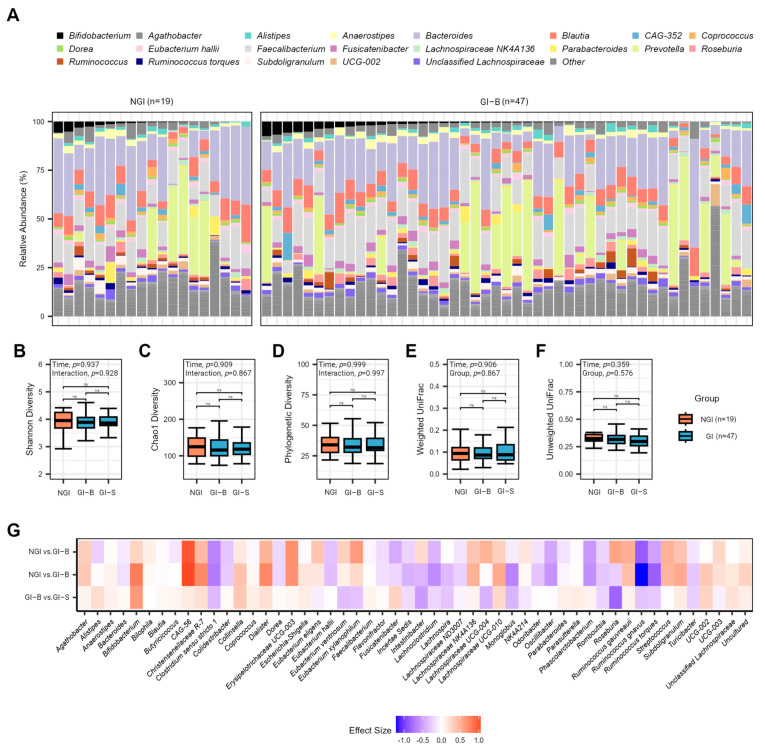
Gut microbiota of NGI and GI subjects (at two different periods listed as GI-B and GI-S). (**A**) Average relative abundance of the most prevalent genera among study subjects at the baseline periods by 16S rRNA sequencing. Genera with a median relative abundance of less than 1% are collapsed into the category “Other.” No significant effects were detected for alpha diversity metrics, metrics, illustrated by *p*-values ≤ 0.05, and pairwise non-significant differences are listed as ns; (**B**) Shannon, (**C**) Chao1, or (**D**) PD diversity. In addition, no significant effects were found for intra-individual distances by time or group for (**E**) weighted UniFrac or (**F**) unweighted UniFrac beta-diversity metrics. (**G**) There were no differentially abundant taxa at the phyla, family, and genera levels between groups for merged baseline periods and day 43 of GI-S (genus level displayed). Effect size displayed as beta-coefficient from ANCOM-BC models.

After establishing that the gut microbiota was generally stable at baseline, we then assessed differences between GI-B when combining day 1 and day 22 with GI-S on day 43. Surveying the taxonomic distribution of merged baseline genus-level abundances, we noted the prominence of Western-dominant gut microbes, like *Bacteroides* and *Blautia*, together with several health-associated microbes, including *Bifidobacterium*, *Faecalibacterium*, *Prevotella*, *Roseburia*, and *Ruminococcus* (Figure 3A).

Assessing alpha diversity via the Shannon index, Chao1, and PD, we observed no significant time (*p* ≥ 0.909) or interaction effects (*p* ≥ 0.867; Figure 3B–D). Moreover, no significant effects of group or time were noted for weighted UniFrac (*p* ≥ 0.840; Supplementary Table S3) or intra-subject distances (*p* ≥ 0.783; Figure 3E). Conversely, the assessment of unweighted UniFrac indicated significant group effects (R^2^ = 0.009, *p* = 0.013; Supplementary Table S4). No significant interaction effects or effects associated with intra-subject distances were found (*p* ≥ 0.359; Figure 3F). Testing homogeneity of dispersion did not reveal any significant differences for UniFrac metrics (*p* ≥ 0.248). Notably, the REAP score had a significant effect within the weighted (*R*^2^ = 0.024; *p* = 0.011) and unweighted UniFrac (*R*^2^ = 0.010; *p* = 0.006) PERMANOVA models. After analyzing 25 REAP components, we found that processed meat, snacking, and sodium-laden foods significantly influenced gut microbiota community composition after a *p*-value correction for UniFrac metrics (q ≤ 0.016; Supplementary Tables S5 and S6). Finally, no microbes at the phyla, family, or genus levels displayed a significant difference between the merged baseline periods for NGI and GI-B and post-intervention for GI-S at our *p*-value correction (q ≤ 0.05; Figure 3G). 

**Figure 4:**
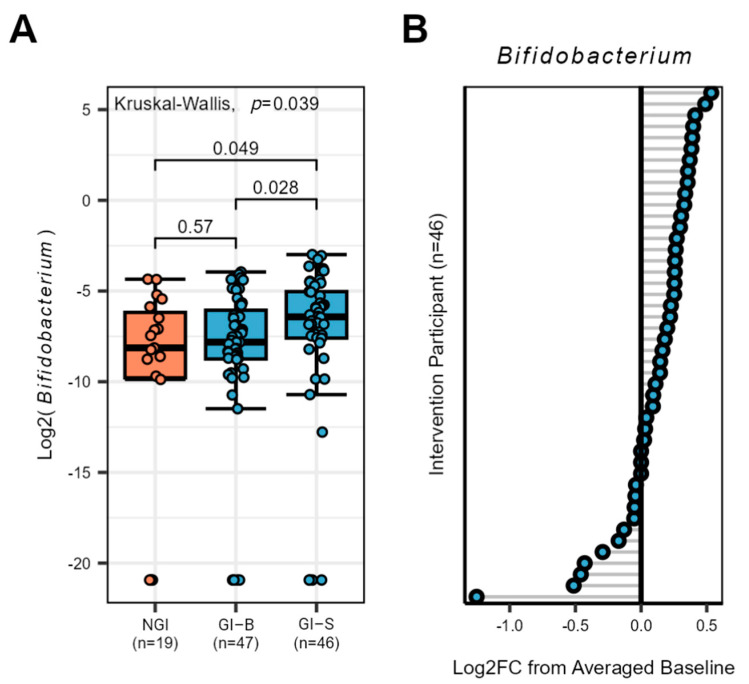
Bifidobacterium response in the gut microbiota of NGI and GI subjects (at two different periods listed as GI-B and GI-S). (**A**) The genus Bifidobacterium increased at day 43 in GI-S subjects compared to the merged baseline period of GI-B, *p*-values are listed for each comparison, and those ≤0.05 were considered significant (**B**) Individualized Bifidobacterium response of GI subjects from the supplementation period (merged baseline GI-B vs. day 43 GI-S), grey lines identify each individual responses illustrated as a circle with blue center.

Despite the lack of significance at the genus level after the *p*-value correction, our a priori hypotheses, anticipating a potential impact of the supplement, led us to independently assess *Bifidobacterium*. No differences were found in the baseline periods of NGI and GI-B, suggesting *Bifidobacterium* stability (*p* ≥ 0.149). After merging the baseline periods, three weeks of supplementation significantly increased levels of *Bifidobacterium* in GI-S compared to GI-B and NGI groups (*p* = 0.028 and *p* = 0.049, respectively, Figure 4A). To better understand the responder status, we assessed individual percent change from baseline to the end of the supplementation period in the intervention group, noting the majority (n = 32) of subjects increased *Bifidobacterium* abundance (Figure 4B).

## 4. Discussion

This study aimed to characterize self-reported GI symptoms in athletes using the GSRS questionnaire, and exploratively assess the effects of a 3-week multi-ingredient fermented whey product on gut microbiota composition, GI complaints, sleep quality, mood, and physical and mental wellbeing in those with self-reported GI complaints. At baseline, a significant difference in GSRS scores and a trend difference in physical wellbeing were observed between the NGI and GI-B groups, but no differences were found for other self-reported secondary outcomes, such as mental wellbeing, sleep quality, and mood disturbance. At baseline, no clear differences in gut microbiota composition were observed between the NGI and GI-B groups. However, after 3 weeks of supplementation, the GI symptoms group exhibited a significant increase in *Bifidobacterium* relative abundance compared to baseline. Notably, this increase in *Bifidobacterium* was observed in the majority of the participants, regardless of GSRS improvement. Furthermore, the supplemented GI symptoms group showed a non-significant trend towards lower GSRS scores and a significant reduction in the number of severe GI complaints compared to its own baseline. 

Self-reported GI complaints at baseline were significantly more prevalent in GI-B (16–94%, with 4–52% severe) vs. NGI (10–52%, with 5–24% severe) groups, for each of the 15 individual GSRS items, in line with the published literature [2,39,61]. However, when GSRS scoring points were analyzed, we found a higher score of 21.1 ± 0.25 for the top-three GI symptoms (bloating, gas/flatus, and hunger pains) compared to Pugh et al., 2018 [2], who reported a prevalence 3 to 9 points lower in elite athletes. Notably in our study, severe but not moderate GI complaints in the GI-B group were significantly reduced (GI-S) after 3 weeks of supplementation. This lack of improvement in moderate complaints could be attributed to the complex nature of GI distress. Indeed, not everyone may respond similarly to interventions [39,62], as we observed a mix of responders and non-responders. Gastrointestinal issues have a multifactorial origin, making them challenging to address and clearly link to a specific physiological disturbance [1,17,25].

In recent years, it has been observed that GI issues in athletes can be associated with microbiota imbalance [25,63], and potentially changes in gut microbiota composition may be necessary to improve these GI symptoms [22,23,24,64]. However, significant improvements in the GSRS over time may not be accompanied by significant changes in gut microbial diversity, as previously described using gut microbiota-targeted interventions [40]. In relation, the lack of major diversity change found in our study might be explained by the relatively healthy and balanced gut microbiota composition found in our subjects. Therefore, the GI distress experienced by the participants may be influenced by factors other than major dysbiosis or alterations in gut microbiota composition. Interestingly, despite the overall lack of major composition changes, we did observe a significant increase in the relative abundance of *Bifidobacterium* after the three-week supplementation period in the majority of the participants. This is consistent with the findings from previous studies investigating the effects of prebiotics or probiotics on gut microbiota composition [65], showing that dietary interventions can lead to a significant increase in *Bifidobacterium* abundance [20,66]. As long as the amount of GOS supplement is high enough [26], the stimulation of *Bifidobacterium* suggests a positive and targeted impact of the supplementation on specific microbial populations within the gut microbiota [27]. Future research should aim to target the gut microbiota and abundance of particular species, such as *Bifidobacterium*, *Lactobacillus*, and *Bacteroides*, as the major contributors to the synthesis of short-chain fatty acids and other health-related gut metabolites. These metabolites may play crucial roles in maintaining gut barrier function, controlling inflammation, and influencing the gut–brain axis [66,67] to prevent or ameliorate exercise induced-GI distress.

The group of athletes with GI symptoms that consumed the multi-ingredient fermented whey product for three weeks showed a significant improvement in self-reported physical wellbeing over time, as assessed by the PHQ questionnaire resulting in lower scores than normal working adults [52] and non-athletic college students [68]. However, no significant improvements were observed on a group level for mental wellbeing, sleep quality, or mood, which is consistent with the literature [36,69,70,71,72,73]. 

As already noted, not every individual seems to benefit from specific bioactive substance interventions [39], while it is possible that the multi-ingredient fermented whey product assessed in this study did not address all the physiological disturbances linked to GI complaints in some participants (i.e., disturbances in gut barrier integrity and/or the occurrence of bacterial translocation and local inflammation) [25]. The exact dosing strategy required to maximize the effect of different bioactive components present in fermented foods is still not well understood [34]. Although there is a growing body of research exploring the gut-health-promoting potential of GOS in human clinical trials [62,74,75,76,77,78], limited literature exists on the prebiotic effects of fermentation metabolites, such as lactic acid and exopolysaccarides [31,32,79], or the postbiotic features of inanimate LGG on gut barrier function and immune health [33], with most studies being in vitro or animal-based. Therefore, more research is needed in humans reporting GI complaints to understand the underlying causes of their symptoms. 

This study has multiple strengths. Notably, it allowed for the comparison of gut microbiota between individuals with self-reported gastrointestinal symptoms and those without such complaints. The study was performed in free-living conditions, while retrospectively showing that neither dietary intake and food quality nor exercise frequency changed over time within groups, confirming that diet and exercise frequency at baseline were comparable between NGI and GI-B individuals. In addition, participants were considered very fit (exercising on average 5–6 times a week), and quick changes in training status is usually limited in trained athletes [80]. However, the study also included some limitations. Individuals were selected based on the self-reporting of GI issues, and no robust clinical test to assess GI complaints independently currently exists. Even athletes in the NGI group reported some complaints after completing the GSRS, indicating that athletes classified themselves as not having GI distress during recruitment, but many of them experienced moderate GI distress to some extent. Furthermore, the inclusion of subjects was not based on other health imbalances, which may have limited the ability to detect clear impacts on mental wellbeing and mood states. Although GSRS scores between the GI group and NGI group were observed to be significantly different at baseline, the small(er) sample size for NGI can be considered a limitation; furthermore, the unequal distribution of males and females did not allow for meaningful sex-based stratifications. Although protein intake was slightly higher in GI-B individuals, absolute protein intake in g/kg did not exceed the current guidelines [81], while the acceptable macronutrient distribution range for macronutrients was not significantly different between groups. Finally, the most important limitation may be that the study had an exploratory character, lacking a control group during the supplementation phase. It is clear that there is a need for well-controlled follow-up studies investigating the relation between self-reported GI complaints, exercise, and diet, as well as investigating the underlying causes of their symptoms. This study provides relevant background insights into this area by characterizing athletes with and without GI symptoms.

## 5. Conclusions

Well-trained athletes with self-reported GI complaints are characterized by more severe GI symptoms when compared to an athletic reference group without self-confirmed symptoms. No differences were seen between groups in baseline microbiota composition. After the three-week consumption of a multi-ingredient fermented whey supplement, *Bifidobacterium* increased in the group receiving the supplement, general wellbeing scores improved, while GI symptoms significantly reduced in severity. Although these changes over time look promising, a randomized controlled trial will be required to confirm and further expand on the causality between these findings and the use of the multi-ingredient whey supplement.

## Figures and Tables

**Table 1:** Demographics for the non-GI (NGI) group without symptoms and the intervention group with symptoms at baseline (GI-B) and after supplementation (GI-S).

Individual Variables	NGI (n = 21)	GI-B and GI-S (n = 50)	*p*-Value
**Age**			
Years	25.7 ± 4.7	26.6 ± 5.4	0.60
**Sex**			
Male, % (n)	52 (11)	66 (33)	0.24
Female, % (n)	48 (10)	32 (16)	
Intersex, % (n)	0 (0)	2 (1)	
**Baseline demographics**			
Age, years (mean ± SD)	26.6 ± 5.42	25.7 ± 4.66	0.60
BMI, kg/m^2^ (mean ± SD)	25.1 ± 3.89	25.1 ± 3.51	0.78
Height, cm (mean ± SD)	171 ± 7.96	174 ± 10.3	0.21
Body weight, kg (mean ± SD)	74.0 ± 15.3	76.6 ± 15.3	0.65
Waist-to-hip ratio (mean ± SD)	0.93 ± 0.20	0.87 ± 0.13	0.25
**Ethnicity**			
Hispanic/Latino, % (n)	14 (3)	18 (9)	0.70
Non-Hispanic/Latino, % (n)	86 (18)	82 (41)	
Race *			
White, % (n)	86 (18)	78 (39)	0.46
Black/African American, % (n)	5 (1)	4 (2)	0.88
Asian/Asian American, % (n)	29 (6)	10 (5)	**0.05**
Native American, % (n)	5 (1)	4 (2)	0.88
Pacific Islander, % (n)	5 (1)	2 (1)	0.52
Indian, % (n)	0 (0)	2 (1)	0.51
Other, % (n)	0 (0)	4 (2)	0.35
Sports *			
Team sports, % (n)	14 (3)	42 (21)	**0.02**
Weight training, % (n)	42 (9)	40 (20)	0.75
Track and field/Endurance, % (n)	19 (4)	12 (6)	0.41
Others, % (n)	24 (5)	6 (3)	**0.01**

* Some subjects identified as mixed race or could be categorized in multiple sports; as a result, the total does not add up to 100%. Significant differences with *p*-values ≤ 0.05 are highlighted in bold.

**Table 2:** Questionnaire-based outcomes for baseline comparison between groups, and difference between baseline (GI-B) and supplementation after day 43 (GI-S) for the GI group, reported as mean ± SD and median (IQR), with F-value and *p*-value.

	Baseline (Day 1 and Day 22)		Comparing Baseline between Groups		Day 43 (after Supplementation)	Comparing Baseline vs. Day 43 in GI Group	
	NGI (n = 21)	GI-B (n = 50)	F-Value	*p*-Value and η^2^	GI-S (n = 50)	F-Value	*p*-Value and η^2^
GSRS (total)	24.1 ± 8.48 21.0 (17.0–29.5)	30.3 ± 8.82 29.8 (23.8–35.6)	**7.46**	**0.008** η^2^ = 0.10	27.2 ± 8.24 25.5 (21.0–30.3)	3.22	*0.076* η^2^ = 0.03
PHQ	33.9 ± 10.7 32.5 (25.3–41.0)	38.5 ± 9.68 38.5 (29.5–45.5)	3.13	*0.081* η^2^ = 0.04	33.3 ± 10.7 32.0 (25.0–42.5)	**6.97**	**0.010** η^2^ = 0.06
K6	4.07 ± 3.79 4.00 (1.00–4.75)	4.67 ± 3.18 4.25 (2.50–6.63)	0.47	0.497 η^2^ = 0.01	3.96 ± 3.60 3.00 (1.00–5.25)	1.11	0.295 η^2^ = 0.01
AIS-8	5.43 ± 4.23 4.00 (3.00–7.50)	5.24 ± 3.69 4.50 (2.50–7.63)	0.03	0.857 η^2^ = 0.001	4.36 ± 3.68 3.00 (1.75–6.25)	1.46	0.230 η^2^ = 0.01
POMS (total mood disturbance)	10.1 ± 16.1 4.00 (−0.75–17.0)	10.4 ± 12.1 8.50 (2.88–14.3)	0.01	0.942 η^2^ < 0.001	9.00 ± 13.3 3.00 (−1.00–16.3)	0.28	0.595 η^2^ < 0.01
REAP (food-quality score)	57.4 ± 4.75 57.0 (54.3–61.5)	57.7 ± 5.43 57.0 (54.8–62.5)	0.06	0.808 η^2^ = 0.001	58.2 ± 6.62 60.0 (53.8–63.0)	0.16	0.687 η^2^ < 0.01
Energy (Kcal—ASA24)	2043 ± 1029 1814 (1268–2309)	2681 ± 1430 2453 (1837–3115)	2.57	0.114 η^2^ = 0.07	2641 ± 1077 2507 (1860–3557)	0.04	0.838 η^2^ < 0.001
Protein (g—ASA24)	101 ± 46.7 84.9 (65.3–129)	157 ± 98.7 125 (97.2–197)	**5.53**	**0.022** η^2^ = 0.13	138 ± 68.5 128 (91.0–169)	1.28	0.261 η^2^ = 0.01
Protein (g/kg)	1.42 ± 0.44 1.26 (1.10–1.77)	1.96 ± 0.80 1.85 (1.28–2.50)	**5.62**	**0.021** η^2^ = 0.08	1.77 ± 0.79 1.72 (1.27–2.17)	1.69	0.198 η^2^ = 0.02
Protein (En%)	21.7 ± 7.31 22.0 (15.0–25.0)	23.1 ± 5.18 22.0 (20.0–25.0)	0.82	0.368 η^2^ = 0.01	21.2 ± 7.12 21.0 (16.0–25.0)	1.91	0.170 η^2^ = 0.02
Fiber (g—ASA24)	16.4 ± 6.60 16.3 (11.0–22.0)	22.2 ± 10.2 22.1 (14.5–27.6)	**4.57**	**0.036** η^2^ = 0.10	22.8 ± 10.3 22.0 (16.2–27.7)	0.05	0.816 η^2^ < 0.01

**Note.** The table presents the following abbreviations: gastrointestinal symptoms rating scale (GSRS), physical health questionnaire (PHQ), Kessler-6 questionnaire for mental wellbeing (K6), Athens insomnia scale (AIS-8), profile of mood states (POMS), and the 24 h recall module providing energy and macronutrient data was ASA-24. Finally, groups were identified as non-GI (NGI) or GI complaints at baseline (GI-B), and after supplementation (GI-S). *p*-values for significance were set at *p* ≤ 0.05 (in bold), but due to the exploratory nature of this study, *p*-values ≤ 0.10 (in italic) were considered trend significant. Additionally, Eta-Squared was calculated as η^2^ = SSeffect/SStotal to examine effect sizes.

**Table 3:** Percentage of gastrointestinal symptoms rating scale (GSRS) scores for individual items for each score on a 1–7 scale at baseline for the non-GI group and GI group, and after supplementation (day 43) for the GI group (score of 1 = no discomfort at all).

	NGI (n = 21)							GI-B (n = 50)							GI-S (n = 50)						
	1	2	3	4	5	6	7	1	2	3	4	5	6	7	1	2	3	4	5	6	7
Upper abdominal pain	81	10	5	10	5	0	0	28	30	16	20	4	2	0	46	32	12	10	0	0	0
Heartburn	90	5	5	5	5	0	0	74	18	2	4	2	0	0	88	6	4	0	2	0	0
Acid reflux	76	14	5	5	5	5	0	64	26	6	0	2	2	0	80	10	8	2	0	0	0
Hunger	52	38	10	10	0	0	0	22	36	20	18	4	0	0	50	24	12	8	6	0	0
Nausea	71	24	10	5	0	0	0	42	38	10	8	0	2	0	68	10	14	6	2	0	0
Rumbling	62	24	14	5	0	0	0	18	38	32	12	0	0	0	38	30	22	6	4	0	0
Bloated	48	24	10	14	10	0	0	12	22	14	30	12	10	0	28	22	22	24	2	2	0
Burping	67	14	12	5	5	0	0	38	34	22	4	2	0	0	60	26	8	4	2	0	0
Gas/flatus	48	19	19	14	5	5	0	6	32	20	24	16	2	0	22	24	32	16	2	0	4
Constipation	71	14	14	5	5	0	0	44	24	16	8	6	2	0	54	14	18	8	6	0	0
Diarrhea	76	10	14	0	5	5	0	50	20	10	14	6	0	0	58	20	12	6	4	0	0
Loose stools	71	14	10	10	0	0	0	66	12	12	8	0	2	0	80	10	8	2	0	0	0
Hard stools	81	10	10	5	0	0	0	54	26	16	2	2	0	0	70	18	6	6	0	0	0
Urgent need to defecate	71	19	5	10	0	0	0	52	28	10	8	0	2	0	66	18	8	4	2	0	2
Incomplete evacuation	67	19	14	10	0	0	0	22	30	22	20	0	6	0	44	22	24	4	4	2	0

**Note.** Abbreviations indicate groups identified as non-GI (NGI) or GI complaints at baseline (GI-B), and after supplementation (GI-S).

## Data Availability

The datasets analyzed during the current study are not publicly available due to data protection regulations. Data of this study may be requested upon an informal inquiry addressed to the corresponding author F.C.W. Each request will then have to pass a formal process of application and review by the sponsor of the study and a scientific board.

## Supplementary Materials

The following supporting information can be downloaded at: https://www.mdpi.com/article/10.3390/nu16111712/s1, Table S1: PERMANOVA model for weighted UniFrac distances comparing groups between baseline periods (Day 1 and 22); Table S2: PERMANOVA model for unweighted UniFrac distances comparing groups between baseline periods (Day 1 and 22); Table S3: PERMANOVA model for weighted UniFrac distances comparing GI-S at Day 43 to GI-B and NGI at the baseline periods (Day 1 and 22); Table S4: PERMANOVA model for unweighted UniFrac distances comparing GI-S at Day 43 to GI-B and NGI at the baseline periods (Day 1 and 22); Table S5: PERMANOVA model for weighted UniFrac distances comparing REAP components across all samples; Table S6: PERMANOVA model for unweighted UniFrac distances comparing REAP components across all samples. Figure S1: Baseline assessment at Day 1 and 22 of the NGI and GI gut microbiota.
