# Supplementary material for: Explorative Characterization of GI Complaints, General Physical and Mental Wellbeing, and Gut Microbiota in Trained Recreative and Competitive Athletes with or without Self-Reported Gastrointestinal Symptoms
Source: Nutrients. 2024 May 30;16(11):1712. doi: 10.3390/nu16111712 (PMC11174857; doi:10.3390/nu16111712)
Supplement: Supplementary file 1 [file nutrients-16-01712-s001.zip › nutrients-3024433-supplementary.pdf]

Supplemental Material

Explorative Characterization of GI Complaints, General Physical and Mental Wellbeing, and Gut Microbiota in Trained Recreative and Competitive Athletes with or without Self-Reported Gastrointestinal Symptoms

1. Supplementary Tables

Table S1. PERMANOVA model for weighted UniFrac distances comparing groups between baseline periods (Day 1 and 22).

	DF	SS	R^2	F	p-value
Group	1	0.0502	0.00955	1.2226	0.268
Time	1	0.0058	0.00111	0.1420	0.998
Sex	1	0.0738	0.01404	1.7973	0.122
WHR	1	0.0156	0.00297	0.3804	0.829
REAP Score	1	0.0412	0.00785	1.0046	0.326
Group*Time	1	0.0175	0.00333	0.4267	0.782
Residual	123	5.0487	0.96114		
Total	129	5.2529	1.00000		

Table S2. PERMANOVA model for unweighted UniFrac distances comparing groups between baseline periods (Day 1 and 22).

	DF	SS	R^2	F	p-value
Group	1	0.2454	0.01070	1.3901	0.053
Time	1	0.0648	0.00282	0.3671	1.000
Sex	1	0.3825	0.01667	2.1665	**0.001**
WHR	1	0.2469	0.01076	1.3987	0.052
REAP Score	1	0.2451	0.01068	1.3880	0.057
Group*Time	1	0.0466	0.00203	0.2637	1.000
Residual	123	21.7163	0.94634		
Total	129	22.9476	1.00000		

Note. Values for significance with a p-value ≤ 0.05 have been highlighted in **bold** font.

Table S3. PERMANOVA model for weighted UniFrac distances comparing GI-S at Day 43 to GI-B and NGI at the baseline periods (Day 1 and 22).

	DF	SS	R^2	F	p-value
Group	1	0.0570	0.00793	1.4029	0.192
Time	2	0.0141	0.00196	0.1735	1.000
Sex	1	0.0854	0.01187	2.1006	0.102
WHR	1	0.0209	0.00290	0.5132	0.679
REAP Score	1	0.1703	0.02368	4.1902	**0.011**
Group*Time	1	0.0161	0.00224	0.3958	0.814
Residual	168	6.8275	0.94942		
Total	175	7.1912	1.00000		

Note. GI-S = group with GI complaints after supplementation, GI-B = the same group with GI complaints at baseline , and NGI = reference group without GI complaints.

Values for significance with a p-value ≤ 0.05 have been highlighted in **bold** font.

Table S4. PERMANOVA model for unweighted UniFrac distances comparing GI-S at Day 43 to GI-B and NGI at the baseline periods (Day 1 and 22).

	DF	SS	R^2	F	p-value
Group	1	0.2773	0.00903	1.6033	**0.013**
Time	2	0.1310	0.00427	0.3787	1.000
Sex	1	0.5042	0.01642	2.9146	**0.001**
WHR	1	0.3482	0.01134	2.0130	**0.001**
REAP Score	1	0.3285	0.01070	1.8992	**0.006**
Group*Time	1	0.0467	0.00152	0.2699	1.000
Residual	168	29.0609	0.94671		
Total	175	30.6969	1.00000		

Note. GI-S = group with GI complaints after supplementation, GI-B = the same group with GI complaints at baseline , and NGI = reference group without GI complaints.
Values for significance with a p-value ≤ 0.05 have been highlighted in **bold** font.

Table S5. PERMANOVA model for weighted UniFrac distances comparing REAP components across all samples.

	DF	SS	R^2	F	p-value	q-value
Skip breakfast	1	0.0520	0.00723	1.5223	0.159	0.289
Eat out	1	0.0273	0.00379	0.7989	0.442	0.480
Whole grains	1	0.1157	0.01609	3.3893	0.029	0.165
Fruit	1	0.0289	0.00402	0.8465	0.427	0.480
Veggies/potatoes	1	0.0858	0.01193	2.5138	0.057	0.178
Milk/yogurt/cheese	1	0.0406	0.00564	1.1888	0.251	0.330
Fat nonfat milk	1	0.0433	0.00602	1.2672	0.237	0.329
Fat low-fat cheese	1	0.0968	0.01346	2.8359	0.045	0.178
Dark meat	1	0.0636	0.00885	1.8638	0.105	0.218
6oz meat per day	1	0.0512	0.00712	1.5001	0.177	0.289
High fat meat	1	0.0334	0.00465	0.9788	0.351	0.417
Skin meat	1	0.0552	0.00768	1.6174	0.163	0.289
Processed meat	1	0.2103	0.02924	6.1602	0.002	**0.016**
Fried foods	1	0.0741	0.01030	2.1694	0.098	0.218
Snacks	1	0.3487	0.04849	10.2155	0.001	**0.016**
Dressing fat	1	0.0519	0.00722	1.5204	0.185	0.289
Butter margarine oil	1	0.0764	0.01063	2.2390	0.082	0.205
Cook oil	1	0.0856	0.01191	2.5087	0.057	0.178
Sweet fat	1	0.0425	0.00591	1.2450	0.220	0.323
Ice cream fat	1	0.1022	0.01421	2.9928	0.033	0.165
Sweets	1	0.0819	0.01138	2.3979	0.080	0.205
Soft drinks	1	0.0382	0.00531	1.1189	0.282	0.352
Sodium food	1	0.2194	0.03050	6.4260	0.002	**0.016**
Add salt	1	0.0201	0.00279	0.5883	0.614	0.614
Alcohol	1	0.0258	0.00359	0.7560	0.473	0.492
Residual	150	5.1205	0.71205			
Total	175	7.1912	1.00000			

Note. Values for significance with a p-value ≤ 0.05 have been highlighted in **bold** font.

Table S6. PERMANOVA model for unweighted UniFrac distances comparing REAP components across all samples.

	DF	SS	R^2	F	p-value	q-value
Skip breakfast	1	0.3216	0.01048	2.0115	0.003	0.289
Eat out	1	0.2568	0.00837	1.6064	0.025	0.480
Whole grains	1	0.2598	0.00846	1.6249	0.013	0.165
Fruit	1	0.2578	0.00840	1.6124	0.021	0.480
Veggies/potatoes	1	0.3662	0.01193	2.2903	0.001	0.178
Milk/yogurt/cheese	1	0.3597	0.01172	2.2497	0.001	0.330
Fat nonfat milk	1	0.2886	0.00940	1.8051	0.004	0.329
Fat low-fat cheese	1	0.2379	0.00775	1.4882	0.024	0.178
Dark meat	1	0.3244	0.01057	2.0287	0.001	0.218
6oz meat per day	1	0.2029	0.00661	1.2688	0.103	0.289
High fat meat	1	0.2230	0.00727	1.3950	0.056	0.417
Skin meat	1	0.2868	0.00934	1.7941	0.006	0.289
Processed meat	1	0.2910	0.00948	1.8202	0.004	**0.016**
Fried foods	1	0.2547	0.00830	1.5932	0.013	0.218
Snacks	1	0.3208	0.01045	2.0062	0.003	**0.016**
Dressing fat	1	0.2947	0.00960	1.8432	0.005	0.289
Butter margarine oil	1	0.2987	0.00973	1.8683	0.003	0.205
Cook oil	1	0.2621	0.00854	1.6394	0.014	0.178
Sweet fat	1	0.2909	0.00948	1.8197	0.006	0.323
Ice cream fat	1	0.2221	0.00723	1.3888	0.050	0.165
Sweets	1	0.1900	0.00619	1.883	0.183	0.205
Soft drinks	1	0.1317	0.00429	0.8234	0.794	0.352
Sodium food	1	0.3208	0.01045	2.0063	0.003	**0.016**
Add salt	1	0.1974	0.00643	1.2346	0.139	0.614
Alcohol	1	0.2542	0.00828	1.5899	0.021	0.492
Residual	150	23.9823	0.78126			
Total	175	30.6969	1.00000			

Note. Values for significance with a p-value ≤ 0.05 have been highlighted in **bold** font.

2. Supplementary Figures

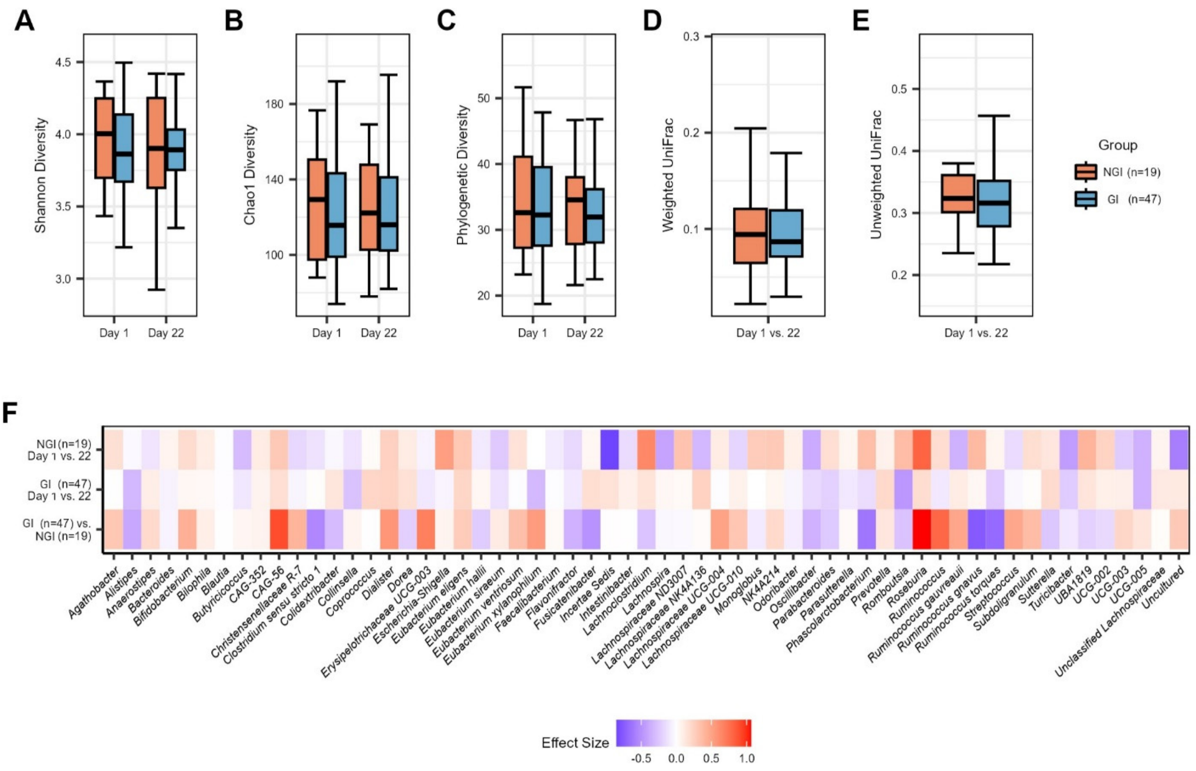

Supplemental Figure S1. Baseline assessment at Day 1 and 22 of the NGI and GI gut microbiota. No significant effects were detected for time or the interaction of group x time for **(A)** Shannon diversity, **(B)** Chao1 diversity, or **(C)** PD diversity. In addition, no significant effects were found for intra-individual distances for **(D)** weighted UniFrac or **(E)** unweighted UniFrac beta diversity metrics. **(F)** There were no differentially abundant taxa at the phyla, family, and genera level between baseline periods and groups (genus level displayed). Effect size displayed as beta-coefficient from ANCOM-BC models.
